# Enhanced microbial degradation of irradiated cellulose under hyperalkaline conditions

**DOI:** 10.1093/femsec/fiaa102

**Published:** 2020-05-27

**Authors:** Naji M Bassil, Joe S Small, Jonathan R Lloyd

**Affiliations:** 1 Research Centre for Radwaste Disposal, Department of Earth and Environmental Sciences, The University of Manchester, Manchester M13 9PL, UK; 2 Williamson Research Centre for Molecular Environmental Science, Department of Earth and Environmental Sciences, The University of Manchester, Manchester M13 9PL, UK; 3 National Nuclear Laboratory, Chadwick House, Birchwood Park, Warrington WA3 6AE, UK

**Keywords:** cellulose irradiation, cellulose hydrolysis, alkaliphiles, hydrogenotrophs, radioactive waste, geological disposal

## Abstract

Intermediate-level radioactive waste includes cellulosic materials, which under the hyperalkaline conditions expected in a cementitious geological disposal facility (GDF) will undergo abiotic hydrolysis forming a variety of soluble organic species. Isosaccharinic acid (ISA) is a notable hydrolysis product, being a strong metal complexant that may enhance the transport of radionuclides to the biosphere. This study showed that irradiation with 1 MGy of γ-radiation under hyperalkaline conditions enhanced the rate of ISA production from the alkali hydrolysis of cellulose, indicating that radionuclide mobilisation to the biosphere may occur faster than previously anticipated. However, irradiation also made the cellulose fibres more available for microbial degradation and fermentation of the degradation products, producing acidity that inhibited ISA production via alkali hydrolysis. The production of hydrogen gas as a fermentation product was noted, and this was associated with a substantial increase in the relative abundance of hydrogen-oxidising bacteria. Taken together, these results expand our conceptual understanding of the mechanisms involved in ISA production, accumulation and biodegradation in a biogeochemically active cementitious GDF.

## Teaser

Microbial degradation of irradiated cellulose can mitigate the ISA-enhanced radionuclide transport from a cementitious geological disposal facility to the biosphere.

## INTRODUCTION

Disposal of radioactive waste in a geological disposal facility (GDF) is considered the safest long-term management solution for these materials (Department for Business, Energy and Industrial Strategy 2018). Different GDF concepts have been proposed, and are under development internationally, that address different geological settings, waste inventories and waste types. All designs implement a multibarrier system that includes physical, chemical and geological containment of radioactive material to impede its transport to the biosphere (Nuclear Decommissioning Authority 2012). Intermediate-level waste (ILW) is defined as being non-heat-generating waste but can include a variety of short-lived β- and γ-emitting radionuclides that generate a high initial dose rate. Longer lived radionuclides present in ILW include actinides (e.g. U, Np, Pu) and metals (Tc, Ni) that have the potential to migrate from groundwater to the biosphere. Concrete materials used in GDF designs for ILW provide a hyperalkaline (pH > 12.5) chemical environment for very long periods (up to 1 million years; Radioactive Waste Management 2016), which promotes the sorption and precipitation of actinides and metals.

Cellulosic materials constitute a substantial portion of the ILW inventory of many countries, arising from routine operations at nuclear power plants and reprocessing facilities (Abrahamsen *et al*. 2015). Typically, these cellulose-containing waste materials are grouted in cement inside steel or concrete canisters for disposal in a cementitious GDF. Under the anaerobic and hyperalkaline GDF conditions, the cellulosic material is expected to undergo chemical hydrolysis (Knill and Kennedy 2003), which produces low-molecular-weight carboxylic acids, together with isosaccharinic acid (ISA; Glaus *et al*. 1999). ISA is a particular concern as it forms water-soluble, alkali-stable complexes with heavy metals and radionuclides (Vercammen, Glaus and Van Loon 2001; Rai *et al*. 2003; Warwick *et al*. 2004; Warwick, Evans and Vines 2006; Evans *et al*. 2008), and thus has the potential to increase radionuclide release to the biosphere. The generation and accumulation of ISA is therefore of critical importance to the assessment of the long-term environmental safety performance of a GDF for ILW.

While there has been significant work to quantify the rate of alkaline cellulose hydrolysis to estimate the formation and accumulation of ISA, and model its potential for radionuclide complexation under ILW disposal conditions (Van Loon and Glaus 1997; Glaus *et al*. 1999; Van Loon *et al*. 1999; Wieland *et al*. 2002; Pavasars *et al*. 2003; Warwick *et al*. 2004; Tits, Wieland and Bradbury 2005; Warwick, Evans and Vines 2006; Glaus and Van Loon 2008), these studies did not address the combined effect of radiolysis and alkali hydrolysis on ISA formation from cellulosic materials. Previous studies have shown that the irradiation of different cellulosic materials (cotton filters, softwood, cotton wool, microcrystalline cellulose) from different radiation sources (electron beam, γ-radiation) led to an increase in the mid-chain scissions and a decrease in the degree of polymerisation of the cellulose (Glegg and Kertesz 1957; Beardmore, Fan and Lee 1980; Bouchard, Méthot and Jordan 2006; Driscoll *et al*. 2009). Since the abiotic hydrolysis of cellulose by alkali propagates by peeling off one glucose subunit from the reducing end of the cellulose polymer (Knill and Kennedy 2003), the increased number of cellulose fibres in the irradiated samples (and therefore the higher number of reducing end glucose units that are exposed to the alkaline environment) is expected to enhance the rate of cellulose hydrolysis by alkali and ISA production.

The hyperalkaline GDF for ILW disposal has been considered an inhospitable environment for microbial colonisation; however, given the size of the GDF and the heterogeneous nature of the wastes, it is likely that lower pH microenvironments that favour microbial growth will develop. A growing body of evidence suggests that microbes can indeed survive under the harsh conditions expected in a cementitious GDF. For example, studies showed that microbes can survive and even thrive under the hyperalkaline conditions that are expected in a cementitious GDF by forming flocks and biofilms that shield them from the high pH (Charles *et al*. 2015, 2017a,b, 2019). Microbes have been found recently to bloom in high doses of ionising radiation in a high-pH outdoor spent nuclear fuel storage pond at Sellafield, UK (Foster *et al*. 2020). Furthermore, we showed in a previous study that the microbial degradation of autoclaved paper tissue under hyperalkaline conditions led to a drop in pH to circumneutral conditions, which caused a stop in ISA production as a result of the abiotic hydrolysis of cellulose by alkali (Bassil *et al*. 2015). Although those samples were inoculated with biologically rich bacterial communities, they were dominated by a few bacterial taxa that are known for their cellulolytic (bacteria belonging to the genus *Clostridia*) and hydrogenotrophic (bacteria belonging to the genus *Sporomusa*) functions after 30 months of incubation (Bassil *et al*. 2015).

This study addresses the gap in our knowledge of the combined effect of irradiation and alkali hydrolysis of cellulose (Kimwipes; Sigma-Aldrich, Dorset, UK), and couples this to the microbial metabolism of the irradiated cellulose. It also extends our understanding of the role that microorganisms may play in a cementitious GDF for ILW, and helps inform more realistic safety cases for a cementitious GDF, where microbes have the potential to mitigate the impact of ISA on radionuclide mobility.

## MATERIALS AND METHODS

### Irradiation

Laboratory-grade tissue paper (Kimwipes; Sigma-Aldrich, Dorset, UK) measuring 5.50 × 5.25 cm^2^ (weighing 0.05–0.06 g) was added to 2 mL of a Ca(OH)_2_ solution (1.5 g/L) in pre-scored 5-mL glass ampules (Wheaton, Millville, NJ, USA), and flame-sealed with air headspace. Some of these ampules were irradiated at a rate of ∼1 kGy/min over a 16-h period and received an average cumulative dose of 1.01 ± 0.3 MGy from a ^60^Co source at the University of Manchester Dalton Nuclear Institute Cumbria Facility, Cumbria, UK. Another set of ampules, which were not irradiated, was stored at room temperature in the dark for the same period of time. This treatment was chosen to represent the cumulative dose received by ILW after several thousand years of storage in the GDF (Abrahamsen *et al*. 2015).

### Sediment

Sediment samples were collected from a depth of ∼20 cm from the surface of a site, at Harpur Hill, Buxton, UK, that had been contaminated for decades by high pH-generating spoil from a legacy lime works (Rout *et al*. 2015b; Smith *et al*. 2016). The sediments at the site have a circumneutral to alkaline pH (pH 12.8 maximum) and contain high calcium and silicate concentrations, analogous to a cementitious radioactive waste repository.

### Microcosm set-up and sampling

Sacrificial microcosms were prepared in 20-mL serum bottles (Agilent, Santa Clara, CA, USA), containing 8 mL of a 1.5 g/L Ca(OH) _2_ solution that was previously flushed with N_2_, 2.5% (w/v) sediment from the high-pH analogue site, and loaded with the contents of the irradiated or unirradiated ampules [the final volume of the microcosms was 10 mL and the Kimwipes constituted 2.5% (w/v)]. These samples were prepared in a Coy anaerobic chamber (Coy Labs, Grass Lake, MI, USA) filled with 2.5% H _2_ and 97.5% N_2_. The bottles were closed with butyl rubber stoppers (Agilent, Santa Clara, CA, USA) and incubated at 20°C for the length of the experiment. Control samples that did not contain the sediment were also prepared. Two replicate samples from uninoculated control bottles and three replicates from the inoculated samples were sacrificed at every time point for headspace analysis by gas chromatography. After analysing the gas phase, the bottles were opened in an anaerobic chamber and the solid and liquid phases were separated by centrifugation at 4000   *g* for 10 min. Aliquots were collected from the supernatants for measurements of pH, the concentrations of ISA, volatile fatty acids (VFA) and dissolved organic carbon (DOC). Aliquots were also taken from the solid phase for light and scanning electron microscopy. The remainder of the liquid and the solid phases were stored at –20°C.

### Analytical measurements

The concentrations of ISA and VFA in all microcosm experiments were determined by ion exchange chromatography as described previously (Bassil, Bryan and Lloyd 2015). The percentages of CH_4_ and H_2_ in the gas phase were measured by gas chromatography as described previously (Kuippers *et al*. 2015). The DOC content in the liquid phase of the microcosm experiments was measured using the high-temperature catalytic oxidation method as described previously (Nixon *et al*. 2018).

### Microscopy

Environmental scanning electron microscopy (ESEM) was used to obtain images of the unirradiated and irradiated Kimwipes. Unirradiated Kimwipe sheets were wetted with deionised water and air-dried overnight on a 12.7 mm diameter top aluminium pin stub (Carl Zeiss Promenade, Jena, Germany). Irradiated Kimwipes (10 μL) were spread on a 12.7 mm diameter top aluminium pin stub (Carl Zeiss Promenade, Jena, Germany) and air-dried overnight. Samples were observed on an FEI XL30 ESEM-Field emission gun (ESEM-FEG; FEI, Hillsboro, OR, USA) operating at 10 kV in high vacuum mode.

Light microscopy was carried out using a Zeiss Axio Imager A1 (Carl Zeiss Promenade, Jena, Germany) light microscope fitted with an Axiocam 506 mono camera, which was controlled by Zen2 imaging software.

### Bacterial and archaeal community analysis

The bacterial and archaeal communities were determined through analysis of the 16S ribosomal ribonucleic acid (rRNA) gene sequences obtained from each replicate of the irradiated and inoculated samples at the different time points of the experiment. Deoxyribonucleic acid (DNA) was extracted from all the samples using the DNeasy PowerSoil Kit (Qiagen, Hilden, Germany) according to the manufacturer's instructions, and quantified using the Qubit dsDNA HS Assay Kit and a Qubit 3.0 Fluorometer (Invitrogen, Paisley, UK; Table S1, Supporting Information).

A small volume (2 μL) from the extracted DNA was used to amplify the V4 hypervariable region of the 16S rRNA gene by polymerase chain reaction (PCR), using a barcoded 515F and 806R primer set (Caporaso *et al*. 2012), and the FastStart High Fidelity PCR System (Roche Diagnostics Ltd, Burgess Hill, UK). A negative control sample was prepared following the same procedure but using sterile water. The PCR was performed in 50-μL reactions under the following conditions: initial denaturation at 95°C for 2 min, followed by 36 cycles of 95°C for 30 s, 55°C for 30 s, 72°C for 1 min and a final extension step of 72°C for 5 min. The barcoded PCR products were purified and normalised to ∼20 ng each, using the SequalPrep Normalization Kit (Fisher Scientific, Loughborough, UK). The normalised, barcoded PCR products were pooled and sequenced on an Illumina MiSeq platform (Illumina, San Diego, CA, USA) using version 2 chemistry to produce 2 × 250-bp pair-end sequencing reads. The sequencing run was performed using a 4 pM sample library spiked with 4 pM PhiX to a final concentration of 10% (Kozich *et al*. 2013). The demultiplexed fastq files produced by the Illumina MiSeq platform were imported and analysed using QIIME 2 version 2019.10 (Bolyen *et al*. 2018). The reads were quality trimmed using the 515F and 806R primer sequences and the Cutadapt plugin (Martin 2011). They were then filtered and denoised using the DADA2 plugin (Callahan *et al*. 2016) via q2-dada2. Taxonomy was assigned to amplicon sequence variants (ASVs) using the q2-feature-classifier (Bokulich *et al*. 2018) classify-sklearn naïve Bayes taxonomy classifier against the Silva 132 99% reference sequences (Quast *et al*. 2012; Yilmaz *et al*. 2014; Glöckner *et al*. 2017).

The extracted DNA was also used for the PCR amplification of the 16S rRNA genes using the 16S Barcoding Kit (SQK-RAB204; Oxford Nanopore Technologies, Oxford, UK) containing the 27F/1492R primer set and the LongAmp Taq 2× Master Mix (New England Biolabs, Ipswich, MA, USA) for sequencing using the Oxford Nanopore MinION platform (Oxford Nanopore Technologies, Oxford, UK). This is a single-molecule sequencer that sequences long DNA fragments as they pass through a nanopore, and therefore can sequence the complete 16S rRNA gene and facilitate more detailed phylogenetic classification of key organisms present in our experiments. The SQK-RAB204 protocol requires the use of 10 ng of extracted genomic DNA; however, the DNA yield was lower than that requirement (Table S1, Supporting Information), and therefore the maximum volume of DNA (24 μL) was added to the PCR mix. A negative control sample was prepared following the same procedure but using sterile water. Amplification was performed as follows: initial denaturation at 95°C for 1 min, 25 cycles of 95°C for 20 s, 55°C for 30 s and 65°C for 2 min, followed by a final extension at 65°C for 5 min. The barcoded PCR products were cleaned using magnetic beads (AMPure XP, Beckman Coulter, High Wycomb, UK), pooled and sequenced on an Oxford Nanopore MinION platform (Oxford Nanopore Technologies, Oxford, UK). The library (75 µL) was loaded in the SpotON port of a R9.4.1 flow cell (Oxford Nanopore Technologies, Ltd) and run using a MinION MK1B device and MinKNOW 2.0. The produced fast5 files were basecalled and demultiplexed, and the barcodes trimmed using Guppy (version 3.4.5). Trimmomatic (version 0.39; Bolger, Lohse and Usadel 2014) was used to trim primer sequences, crop the initial 50 bp and any bases longer than 1500 bp and retain reads that are longer than 1100 bp. The resultant fastq files were imported and analysed using QIIME 2 version 2019.10 (Bolyen *et al*. 2018). The VSEARCH plugin (Rognes *et al*. 2016) was then used to dereplicate the sequences, identify and remove chimeras, and cluster the reads at 85% identity using open-reference clustering and the Silva 132 99% reference sequences (Quast *et al*. 2012; Yilmaz *et al*. 2014; Glöckner *et al*. 2017). Taxonomy was assigned to the produced operational taxonomic units (OTUs) using the q2-feature-classifier (Bokulich *et al*. 2018) classify-sklearn naïve Bayes taxonomy classifier against the Silva 132 99% reference sequences (Quast *et al*. 2012; Yilmaz *et al*. 2014; Glöckner *et al*. 2017).

The OTU, ASV and taxonomy tables produced from the analysis of the reads from the two sequencing platforms were imported into R (version 3.6.1) using the package qiime2R (version 0.99.12) (Bisanz 2018). Plots of Shannon diversity index, and principal coordinate analysis (PCoA) on Bray–Curtis dissimilarity matrixes, were produced using the packages phyloseq (version 1.28.0) (McMurdie and Holmes 2013) and vegan (version 2.5–6) (Oksanen *et al*. 2019).

The representative sequences that were assigned to the *Caldicoprobacter* genus were used to search for phylogenetic neighbours with validly published names using the EzBioCloud identification service (http://www.ezbiocloud.net/; Yoon *et al*. 2017). These sequences were aligned to available sequences of all type strains with validly published names belonging to the genera *Caldicoprobacter*, *Pertoclostridium*, *Caloranarobacter* and *Alkaliphilus* using Clustal W (Thompson, Higgins and Gibson 1994) in the software package MEGA version X (Kumar *et al*. 2018; Stecher, Tamura and Kumar 2020). A phylogenetic tree was reconstructed with the neighbour-joining algorithm (Saitou and Nei 1987) using maximum composite likelihood distance (Tamura, Nei and Kumar 2004) with the pairwise deletion option (1440 positions in final dataset), gamma distributed with an invariant site value of 5 and bootstrap (Felsenstein 1985) of 1000 replications.

### Data availability

Sequence data are available in the NCBI Sequence Read Archive database under project accession number PRJNA558914.

## RESULTS

### Irradiation enhances the rate of the abiotic cellulose hydrolysis by alkali

**Figure 1.**
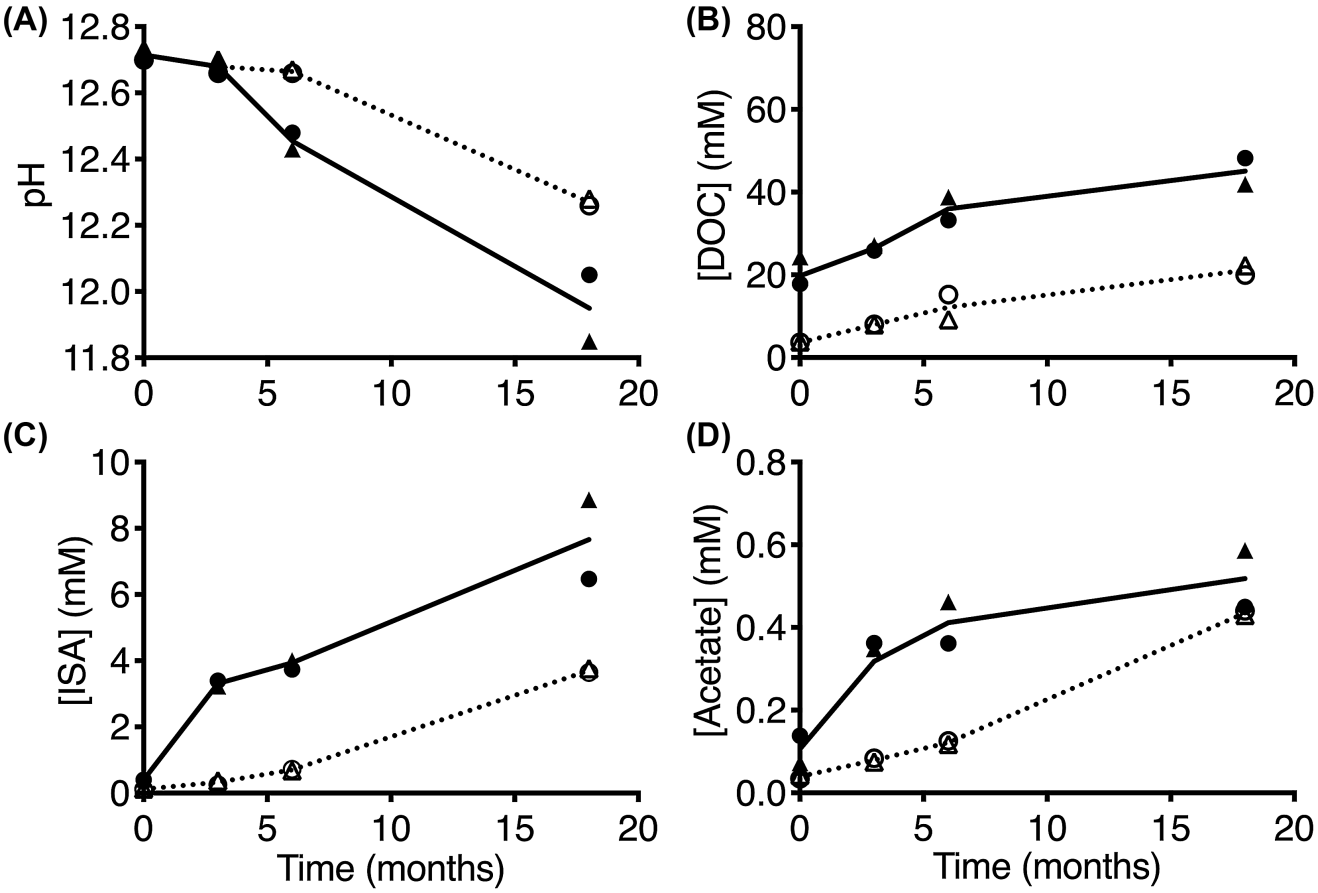
Cellulose irradiation under hyperalkaline conditions enhanced the production of ISA over time via the abiotic alkali hydrolysis of cellulose. **(A)** The pH of the solution. **(B)** The concentration of DOC in solution in mM. **(C)** The concentration of ISA in solution in mM. **(D)** The concentration of acetic acid in solution in mM. Open symbols represent unirradiated samples, and closed symbols represent irradiated samples. Solid line represents the average of the irradiated samples, and dashed line represents the average of the unirradiated samples. • represents replicate 1 in the set and ▲ represents replicate 2 in the set.

Irradiation of cellulosic material in air or water has been previously shown to increase the number of mid-chain scissions and decrease the degree of polymerisation of the cellulose (Glegg and Kertesz 1957; Beardmore, Fan and Lee 1980; Bouchard, Méthot and Jordan 2006; Driscoll *et al*. 2009). This current work suggests that similar processes occur when cellulosic material (Kimwipes) is irradiated with 1 MGy of γ-radiation under hyperalkaline conditions [in a saturated Ca(OH)_2_ solution]. The irradiated cellulose sheets were disaggregated compared to the unirradiated sheets (Figure S1, Supporting Information), and the colour of the sheets and the surrounding solution turned to reddish-orange post-irradiation (Figure S1, Supporting Information). Light and scanning electron microscopy showed that the cellulose fibres were much shorter in the irradiated material compared to the unirradiated ones, indicating that irradiation resulted in an increase in mid-chain scissions in the cellulose fibre (Figure S2, Supporting Information). As expected, this resulted in an increase in the rate of ISA and acetate production (due to the increase in the amount of reducing end groups that the hydroxide can react with, and therefore increases the rate of cellulose hydrolysis), and a more rapid drop in pH over time in the irradiated and uninoculated samples as compared to the unirradiated and uninoculated controls (Fig. 1). The pH decreased from a pH value of 12.7 ± 0.1 to an average pH value of 12.0 ± 0.1 after 18 months of incubation in the irradiated and uninoculated samples (Fig.   1A, solid line), while it dropped to an average pH value of 12.3 ± 0.1 in the unirradiated and uninoculated controls under the same incubation conditions (Fig.   1A, dotted line). These samples also showed an increase in DOC from an average value of 19.8 ± 3.3 mM to an average value of 45.1 ± 3.2 mM in the irradiated and uninoculated samples (Fig. 1B, solid line), and from an average value of 2.0 ± 0.1 mM to an average value of 21.1 ± 1.1 mM in the unirradiated and uninoculated controls (Fig.   1B, dotted line). It is important to note that irradiation of the paper tissue under hyperalkaline conditions caused a significant increase in the DOC content prior to incubation, and this was not due to an initial release of ISA or VFAs into solution (Fig. 1B–D). This initial increase in DOC may be the result of the release of soluble organic molecules including sugars (for example, glucose, cellobiose and xylose), aldehydes (for example, formaldehyde, acetaldehyde and malonaldehyde) and ketones (for example, acetone), which have been reported previously as radiolysis products of cellulose under neutral pH conditions (Bluďovský, Procházka and Kopoldová 1984). These would not have been detected in these measurements because the ion exchange chromatography system utilised in this study is set up for the detection of organic acids only. Irradiation of the paper tissue led to a more rapid production of ISA and acetate over the incubation period post-irradiation, reaching an average concentration of 7.7 ± 1.2 and 0.5 ± 0.1 mM, respectively, in the irradiated and uninoculated samples (Fig. 1C and D, solid lines). However, the average ISA and acetate concentrations were 3.7 ± 0.1 and 0.4 mM, respectively, in the unirradiated and uninoculated controls after 18 months of incubation (Fig. 1C and D, dotted lines). These results were expected given that irradiation increases the number of mid-chain scissions in the cellulose fibres and exposes more reducing end glucose molecules to the alkali, which enhances the rate of cellulose degradation.

### Irradiation increases cellulose bioavailability for bacterial degradation

**Figure 2.**
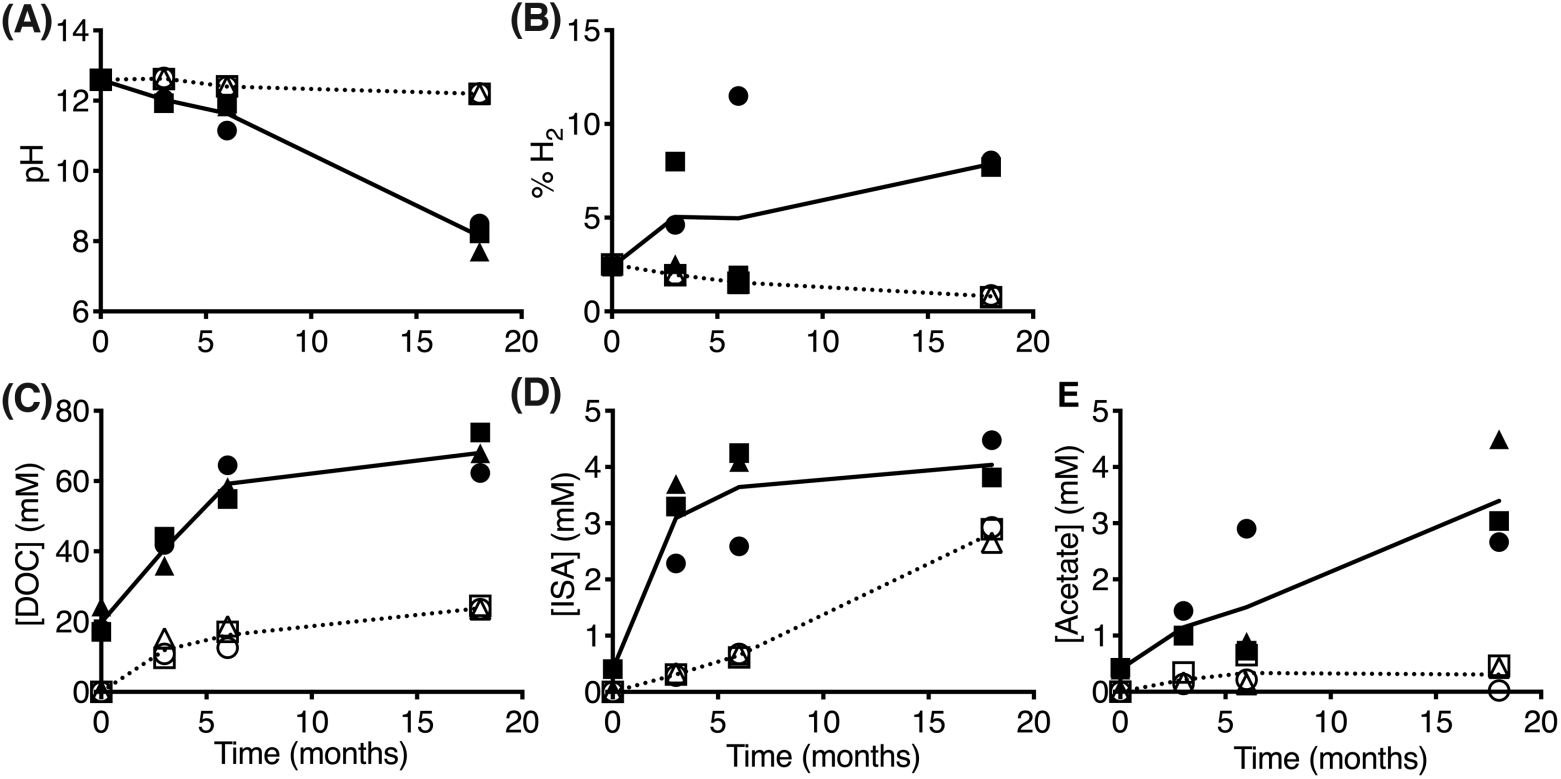
Cellulose irradiation under hyperalkaline conditions made the cellulose polymers more available for microbial degradation and the fermentation of the degradation products, produced acetic acid, and hydrogen, and causes a stop in ISA production. **(A)** The pH of the solution. **(B)** The percentage of hydrogen in the headspace. **(C)** The concentration of DOC in solution in mM. **(D)** The concentration of ISA in solution in mM. **(E)** The concentration of acetic acid in solution in mM. Open symbols represent unirradiated samples and closed symbols represent irradiated samples. Solid line represents the average of the irradiated samples and dashed line represents the average of the unirradiated samples. • represents replicate 1 in the set, ▲ represents replicate 2 in the set and ▓ represents replicate 3 in the set.

The increase in the number of exposed cellulose ends is also expected to increase the availability of cellulose to cellulolytic enzymes, particularly cellobiohydrolases that hydrolyse the ends of the cellulose molecule and release cellobiose (two glucose subunits that are linked by a *β*-1,4-glycosidic bond) for further degradation by *β*-glucosidases, which release glucose molecules for further degradation (Mosier *et al*. 1999). Indeed, the irradiated and inoculated samples showed the accumulation of hydrogen gas in the headspace (up to 11% of the headspace content), and a rapid increase in the DOC (average concentration of 68.0 ± 3.3 mM) and acetate concentrations (average concentration of 3.4 ± 0.6 mM), as a result of the microbial degradation of cellulose and the fermentation of the degradation products (Fig. 2, solid lines). These microbial processes also presumably led to the production of CO_2_, which in addition to the production of acetate and other organic acids, led to a drop in the pH of the solution (due to the formation of carbonic acid) to circumneutral pH conditions (Fig. 2A, solid line). The drop in pH in these samples caused a cessation of ISA production, where although the concentration of ISA increased rapidly during the initial 3 months of incubation, it stabilised at an average concentration of 4.0 ± 0.2 mM for the remainder of the experiment (Fig. 2D, solid line). In contrast, the unirradiated and inoculated controls showed little microbial activity, with only a slight drop in pH to a pH value of 12.0 ± 0.1, along with a decrease in the hydrogen gas content of the headspace, and a slight increase in the DOC and acetate concentrations to reach an average concentration of 23.9 ± 0.1  and 0.5 mM, respectively, over the incubation period (Fig.   2, dotted lines). As expected, a continuous production of ISA was observed in these samples as the pH remained alkaline, and it showed a similar trend to the unirradiated and uninoculated controls (Fig.   1C, dotted line), and reached an average concentration of 2.8 ± 0.1 mM after 18 months of incubation (Fig. 2D, dotted line). This complements previous work that showed that irradiation induced mineralogical changes that increased the bioavailability of synthetic Fe(III) minerals in laboratory cultures (Brown *et al*. 2014) and also stimulated Fe(III) reduction in more complex sediment microcosms (Brown *et al*. 2015).

These chemical changes, especially the changes in the pH of the solution over the incubation period, correlated with changes in the bacterial communities that dominated the irradiated and inoculated samples. Exposure of the background sediment to high pH (pH values > 12.5) for prolonged periods (up to 6 months) led to a drop in the bacterial and archaeal diversity, as evidenced by the drop in the Shannon index (Figure S3A, Supporting Information), and the clustering of the replicates at 6 months in the PCoA plot (Figure S3B, Supporting Information). The bacterial and archaeal diversity later recovered slightly as the pH reached circumneutral pH, and the conditions became more favourable for neutrophilic microbes that had survived the initial high pH to dominate the samples after 18 months of incubation (Figure S3, Supporting Information). The PCoA plot also showed a clear separation between the datasets from the two sequencing platforms utilised in this study (Figure S3B, Supporting Information), which is in line with previous observations that sequencing platforms, library preparation protocols, the primer set used for PCR amplification and the bioinformatics pipeline used contribute to the differences observed in microbial diversity and species richness (Tremblay *et al*. 2015; Allali *et al*. 2017; Winand *et al*. 2019).

**Figure 3.**
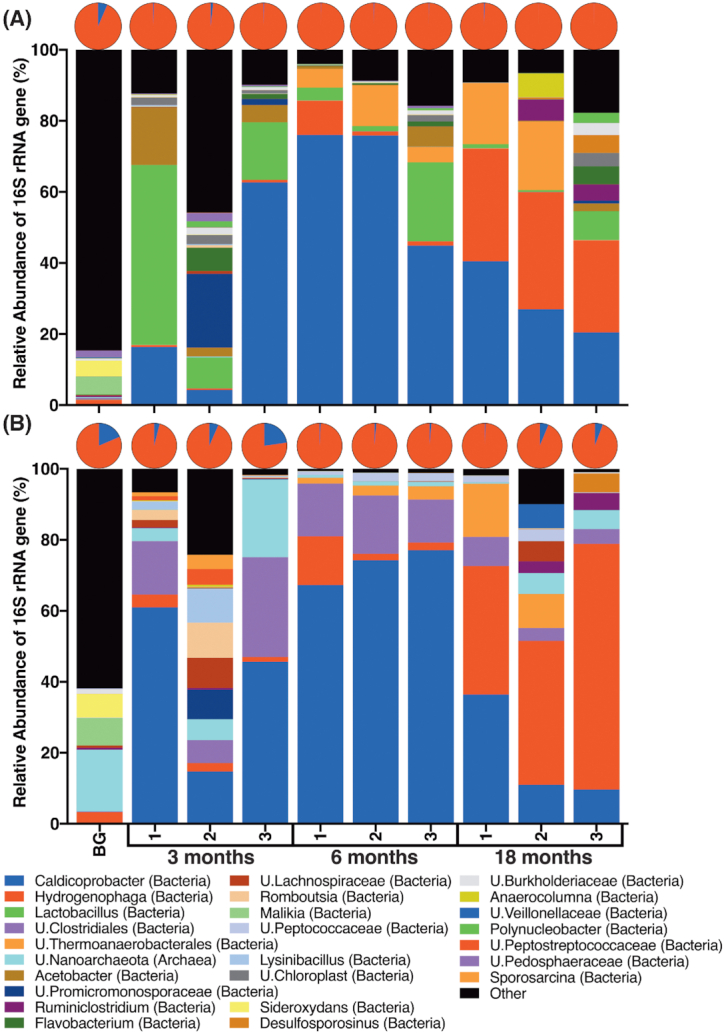
Microbial community analysis of the 16S rRNA gene sequences produced using the Illumina MiSeq platform **(A)**, and the Oxford Nanopore MinION platform **(B)**. Pie charts represent the phylogenetic classification at the Kingdom level, showing Archaea in blue and Bacteria in red. The column charts represent the microbial community structure at the genus level, showing dominance of bacteria belonging to the *Caldicoprobacter* genus in the majority of samples. The vertical axis represents the percentage of the 16S rRNA gene reads assigned to each taxonomic group. The horizontal axis represents the replicate samples at the different time points in the order, background sediment sample, the three replicate samples after 3 months of incubation, the three replicate samples after 6 months of incubation and the three replicate samples after 18 months of incubation.

Differences were also observed in the taxonomic classification of a few ASVs/OTUs that dominated the samples (Fig. 3). The background sediment sample showed higher bacterial and archaeal diversity, and the presence of more archaeal ASVs than the incubated microcosm samples, and these were more evident in the MinION analysis (19%) compared to the MiSeq analysis (6.5%) (Fig. 3). The incubated microcosms showed heterogeneity between the replicate samples, especially during the initial 3 months of incubation (Fig. 3). For example, replicate 1 was dominated by ASVs assigned to the *Lactobacillus* genus (51%) in the MiSeq analysis and OTUs assigned to the *Caldicoprobacter* genus (61%) in the MinION analysis. Replicate 2 at this time point did not show dominance by any particular bacterial genus, while replicate 3 was dominated by ASVs/OTUs assigned to the *Caldicoprobacter* genus (63 and 46%, respectively) in both analyses. Archaea constituted 0.4–1.7% of the ASVs/OTUs in the samples at this time point in the MiSeq analysis, and 4–23% in the MinION analysis, indicating that some Archaea may have survived the high-pH (pH values > 11.9) conditions during the initial 3 months of incubation. After a prolonged period (6 months) of incubation at high pH (pH value > 11.2), the bacterial community in the triplicate samples became dominated by ASVs/OTUs assigned to the genus *Caldicoprobacter* in both the MiSeq (76, 76 and 45%) and the MinION analyses (67, 74 and 77%). The relative abundance of ASVs/OTUs assigned to Archaea dropped to 0.2–1.4%, indicating that the majority of Archaea did not survive at high pH for a prolonged period of time. After 18 months of incubation (when the pH had become circumneutral and hydrogen was detected in the headspace of all the replicate samples), the bacterial communities were dominated by bacteria belonging to the *Hydrogenophaga* genus, members of which are known to oxidise hydrogen (Willems *et al*. 1989); these were identified in the MiSeq (32, 33 and 26%) and MinION (36, 41 and 69%) analyses, albeit in different ratios. Although the MiSeq analysis showed a further drop in the relative abundance of ASVs assigned to Archaea in these replicates (0.1–0.8%), the MinION analysis showed a recovery of the Archaea community (0.6–6.3% of the OTUs).

Identification of ASVs/OTUs to the genus *Caldicoprobacter* is interesting, especially as all isolated species of this genus are thermophilic bacteria that grow at a much higher temperature (45–85°C) and lower pH (5–9 pH units) than those implemented in these experiments (Bouanane Darenfed *et al*. 2011, 2013; Ben Hania *et al*. 2015). Two representative sequences were assigned to this genus in the MinION analysis (sequence length 1215 and 1500 bp) and one in the MiSeq analysis (sequence length 253 bp). These showed 84–86% alignment identity to 16S rRNA gene sequences of type strains of this genus, which were also similar to alignment identities to 16S rRNA gene sequences of type strains of other genera (84–88%), including *Caloranaerobacter*, *Petroclustridium* and *Alkaliphilus*. These low alignment identities, the inability of known *Caldicoprobacter* strains to grow under the experimental conditions implemented in this study and the clear clade separation of the representative sequences assigned to this genus in the neighbour-joining phylogenetic tree (which is based on the almost complete 16S rRNA gene sequence determined through the MinION analysis; Figure S4, Supporting Information) indicate that these sequences may represent a novel bacterial genus that is related to the *Caldicoprobacter* and *Petroclustridium* genera within the family Clostridiaceae. Members of this Gram-positive family are well known to degrade cellulose under high-pH conditions and produce H_2_ and CO_2_ from the fermentation of the degradation products (Zhilina *et al*. 2005; Ren *et al*. 2007).

## DISCUSSION

The pH in a cementitious GDF is expected to be hyperalkaline for a long period of time, which will induce abiotic hydrolysis of the cellulosic material in the ILW and release radionuclide complexants like ISA (Glaus *et al*. 1999; Askarieh *et al*. 2000; Knill and Kennedy 2003). Here, we showed that irradiation of cellulosic material with 1 MGy of γ-radiation under hyperalkaline conditions [in a saturated Ca(OH)_2_ solution] enhanced the rate of cellulose hydrolysis by alkali, and accelerated ISA production consistent with increasing the number of mid-chain scissions in the cellulose fibres, which would expose reducing end glucose units to hydroxide in the solution.

The GDF and the ILW deposited in it are expected to be heterogeneous, which enables the formation and development of lower pH microenvironments that favour microbial growth. Here, we showed that microbial processes in the samples containing irradiated tissue led to the production of hydrogen gas, acetate and a drop in the pH of the solution (due to the possible formation of carbonic acid), which caused a stop in the production of ISA as an alkali hydrolysis product of cellulose. It is plausible that the organics released into the solution as a result of cellulose irradiation (initial increase in DOC before the initiation of incubation) were utilised by microorganisms to initiate colonisation of the samples. This may also explain the relatively high bacterial and archaeal diversity in the samples after 3 months of incubation under hyperalkaline pH conditions, which dropped after 6 months of incubation and the bacterial and archaeal ASVs/OTUs became dominated by alkaliphilic/alkalitolerant, cellulolytic microbes (belonging to the Clostridiaceae family) as cellulose became the only available source of electrons. After 18 months of incubation, when the pH became circumneutral, neutrophilic microbes that potentially survived the initial high-pH conditions and can utilise the by-products of glucose fermentation (for example, members of the *Hydrogenophaga* genus) dominated the samples.

Microbial processes could influence the performance of the GDF as a whole, for example, extremophilic microorganisms that could grow under GDF-similar conditions may play a significant role in reducing radionuclide transport to the biosphere through (i) the direct or indirect bioreduction of radionuclides, (ii) biomineralisation into insoluble solid phases or (iii) bioaccumulation into or biosorption onto the surface of microorganisms (Newsome, Morris and Lloyd 2014). In addition, this and previous studies (Rout *et al*. 2014, 2015a, 2015b; Bassil *et al*. 2015; Bassil, Bryan and Lloyd 2015; Kuippers *et al*. 2015) also suggest that the biodegradation of cellulosic materials and their alkaline hydrolysis products (for example, ISA) may nullify the complexation of radionuclides by organics, and therefore mitigate the enhanced solubility and mobility of the organic–radionuclide complex (Nuclear Decommissioning Authority 2010). Microbial fermentation of the organic material and the accumulation of acetate, hydrogen and carbon dioxide may lead to a drop in pH and to over-pressurisation of the GDF, which is in line with observations by Beaton, Pelletier and Goulet (2019) at lower pH conditions; however, these will be mitigated by the use of porous cementitious grout materials, which have been developed to absorb CO_2_ gas and manage the generation of other gases (for example, H_2_) generated by steel corrosion (Leupin *et al*. 2016; Radioactive Waste Management 2016). The accumulation of acetate, hydrogen and carbon dioxide may induce methane production through acetoclastic or hydrogenotrophic methanogenesis; however, methane and methanogenic Archaea were not detected after 18 months of incubation in this study and after 30 months of incubation in a previous study (Bassil *et al*. 2015). Furthermore, the identification of hydrogenotrophic bacteria (bacteria belonging to the genera *Hydrogenophaga* in this study and *Sporomusa* in Bassil *et al*. 2015) along with the significant drop in the percentage of 16S rRNA gene sequences assigned to Archaea suggest that methanogenic archaea may not survive the prolonged period of hyperalkaline conditions expected to dominate in the GDF.

Generally, comparable results were obtained for the dominant genera from analysis of the MiSeq-generated and the MinION-generated datasets. As expected, more archaeal OTUs were detected, and genus-level identification of the dominant representative sequences was attained by the MinION-produced reads; however, this came at the cost of a lower number of reads that were not uniformly distributed between the samples. This is primarily due to the sequencing strategies employed by this technology that does not utilise amplification during sequencing, and therefore requires much higher concentrations of better-quality DNA. This is in line with other studies comparing between the MiSeq and the MinION platforms for 16S rRNA gene sequencing (Edwards *et al*. 2019; Winand *et al*. 2019; Nygaard *et al*. 2020).

This and previous studies highlight the importance of understanding the interplay between the abiotic (alkali hydrolysis and radiolysis) and biological degradation of cellulose (and the produced ISA), which could influence the accumulation of ISA in the GDF, its availability for radionuclide complexation and mobilisation, and therefore the performance of the GDF. These results also highlight the need to characterise the microbial ecology of these high-pH environments more thoroughly, for example, through shotgun metagenomics and culture-based isolation, to build more complete databases to help better identify major individuals in these high-pH microbial communities and understand their potential roles in these environments.

## Supplementary Material

fiaa102_Supplemental_File
