# Supplementary material for: Enhanced microbial degradation of irradiated cellulose under hyperalkaline conditions
Source: FEMS Microbiol Ecol. 2020 May 27;96(7):fiaa102. doi: 10.1093/femsec/fiaa102 (PMC7329180; doi:10.1093/femsec/fiaa102)
Supplement: fiaa102_Supplemental_File [file fiaa102_supplemental_file.docx]

**Enhanced Microbial Degradation of Irradiated Cellulose Under Hyperalkaline Conditions**

Naji M. Bassil,^1,2*^ Joe S. Small,^1,3^ Jonathan R. Lloyd^1,2^

^1^Research Centre for Radwaste Disposal, Department of Earth and Environmental Sciences, The University of Manchester, Manchester, UK

^2^Williamson Research Centre for Molecular Environmental Sciences, Department of Earth and Environmental Sciences, The University of Manchester, Manchester, UK

^3^National Nuclear Laboratory, Chadwick House, Birchwood Park, Warrington, UK

**Supplementary Table 1**: Summary of the sequencing data associated with the different samples.

|  | **Extract** | **MiSeq** | | | | **MinIon** | | | |
| --- | --- | --- | --- | --- | --- | --- | --- | --- | --- |
| **Sample** | **[DNA] (ng/mL)** | **[amplified DNA] (ng/mL)** | **Number of Reads** | **Read Length (Mean±SD)** | **Number of Reads after QC** | **[amplified DNA] (ng/mL)** | **Number of Reads** | **Read Length (Mean±SD)** | **Number of Reads after QC** |
| **Background sediment** | 0.516 | 0.755 | 105035 | 251±1 | 72783 | 0.683 | 352789 | 1463±287 | 301846 |
| **3_months_1** | 0.23 | 0.604 | 133503 | 251±1 | 86934 | 0.381 | 43295 | 1581±212 | 40949 |
| **3_months_2** | 0.164 | 0.897 | 122162 | 251±1 | 97147 | 0.183 | 38575 | 1424±437 | 32768 |
| **3_months_3** | 0.429 | 0.799 | 122605 | 251±1 | 93241 | 0.537 | 90603 | 1575±243 | 83716 |
| **6_months_1** | 0.3 | 0.774 | 105890 | 251±1 | 85688 | 0.388 | 107287 | 1580±219 | 100630 |
| **6_months_2** | 0.288 | 0.906 | 105044 | 251±1 | 85578 | 0.318 | 69447 | 1593±210 | 65692 |
| **6_months_3** | 0.235 | 1.01 | 105325 | 251±1 | 81311 | 0.233 | 48589 | 1601±204 | 46325 |
| **18_months_1** | 0.079 | 1.22 | 146335 | 251±1 | 106792 | 0.065 | 6912 | 1458±396 | 6083 |
| **18_months_2** | 0.448 | 1.07 | 133088 | 251±1 | 99364 | 0.419 | 153330 | 1565±228 | 143150 |
| **18_months_3** | 0.277 | 0.781 | 118083 | 251±1 | 83937 | 0.355 | 59190 | 1553±204 | 56177 |
| **Negative control** | - | 0.05 | 119 | 251±1 | 21 | - | 35 | 1273±421 | 24 |


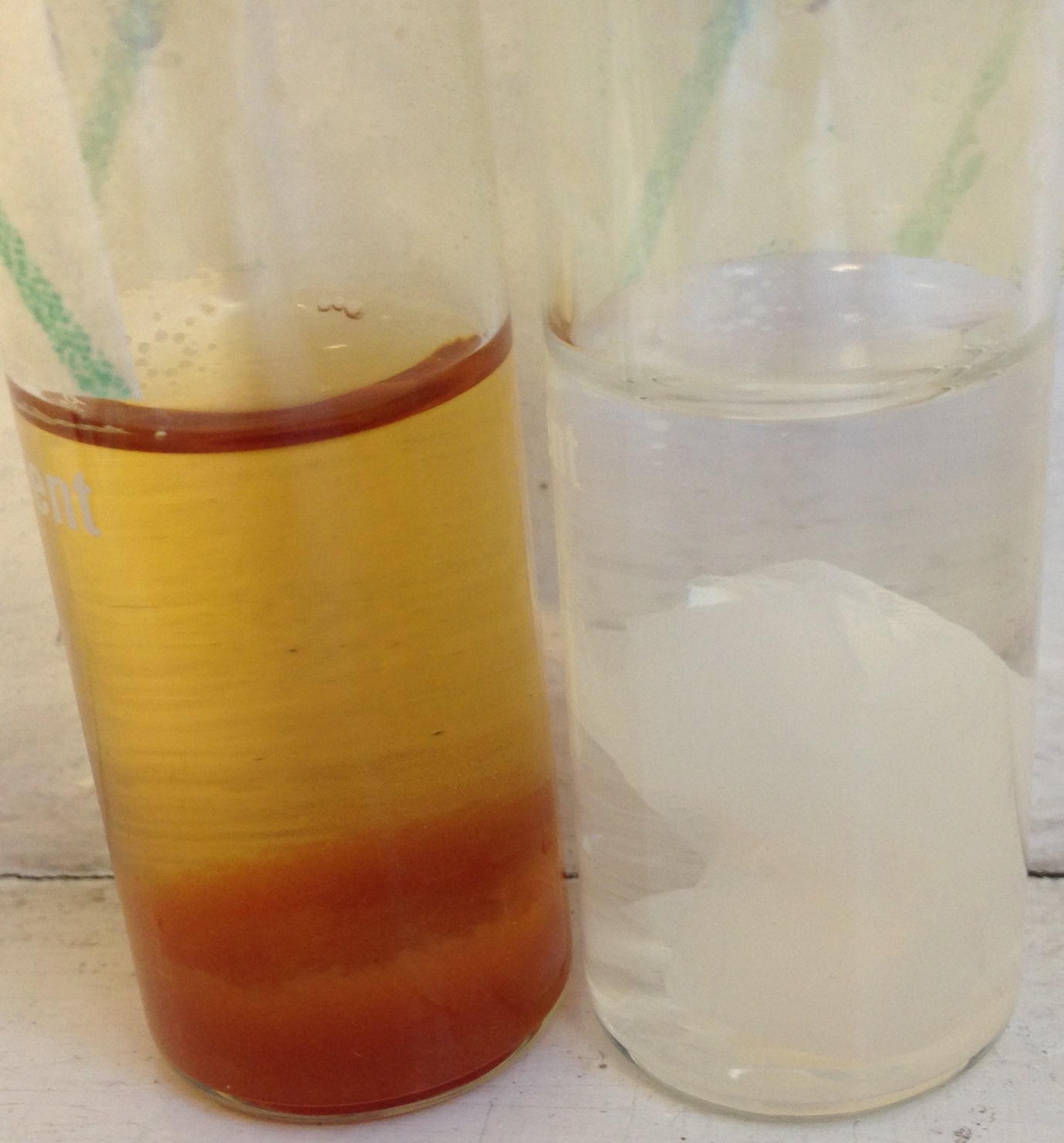


**Supplementary Figure 1:** Picture showing the change in colour of the Kimwipes and the solution post-irradiation (left), as compared to unirradiated Kimwipes (right) in a saturated solution of Ca(OH)_2_ (1.5 g/L).

**
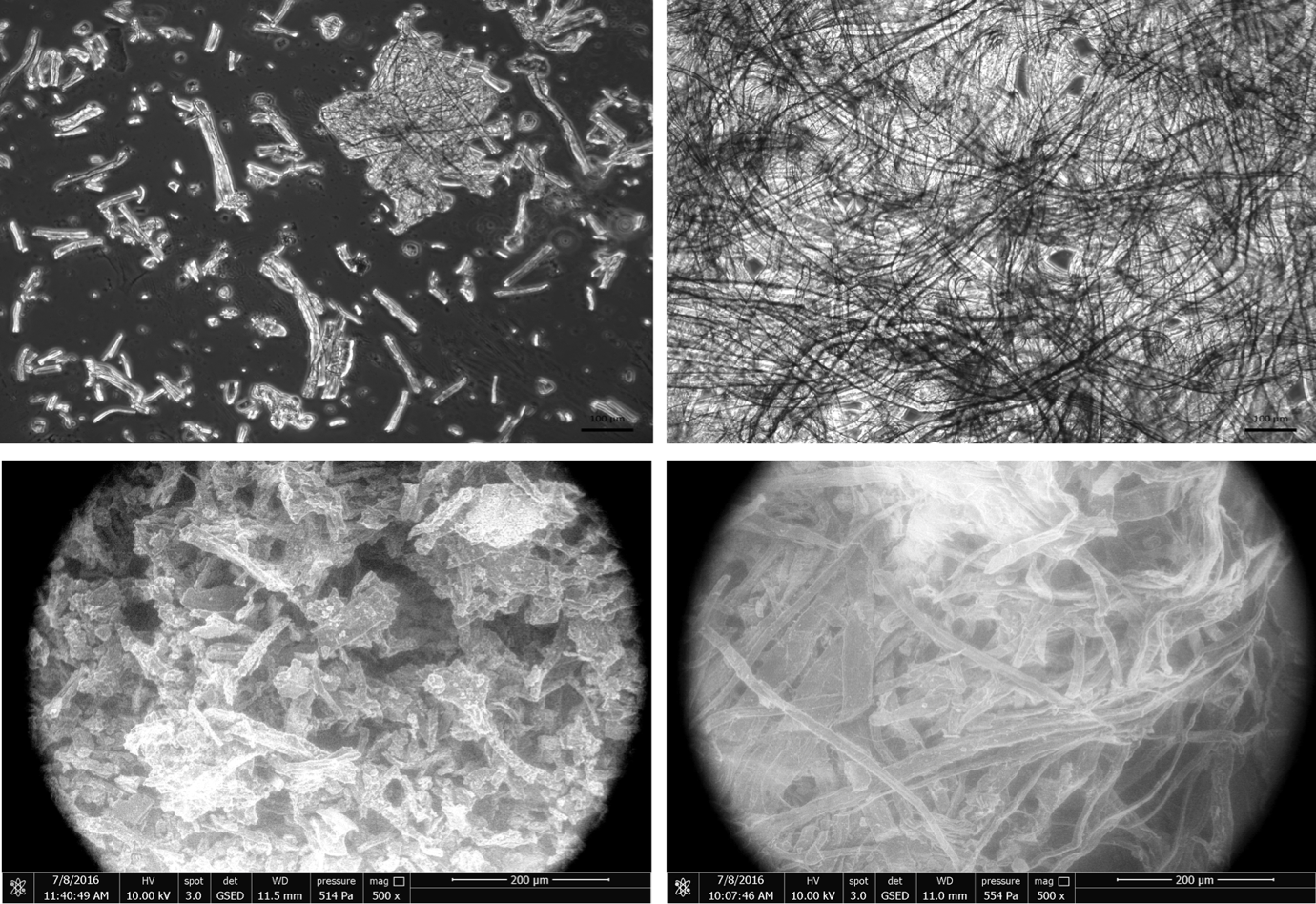
**

**Supplementary Figure 2:** Light microscope image of irradiated (top left) and unirradiated (top right) Kimwipes at 100x magnification (bar represents 100 μm), and ESEM images of irradiated (bottom left) and unirradiated (bottom right) Kimwipes at 500x magnification (bar represents 200 μm).

 **Supplementary Figure 3:** A) Shannon diversity index of the background sediment samples and the replicate samples at 3, 6 and 18 months of incubation, where red represents replicate 1 in the set, blue represents replicate 2 in the set, and green represents replicate 3 in the set. Left panel represents the Shannon diversity of the MinION-produced reads, and the right panel represents the Shannon diversity of the MiSeq-produced reads. B) Principal coordinate analysis (PCoA) plot with Brey-Curtis algorithm of bacterial community structure from the background sediment (red), and the replicate samples at 3 (green), 6 (blue), and 18 (purple) months of incubation, where ● represents the dataset from the MiSeq analysis, and ▲ represents the dataset from the MinION analysis.


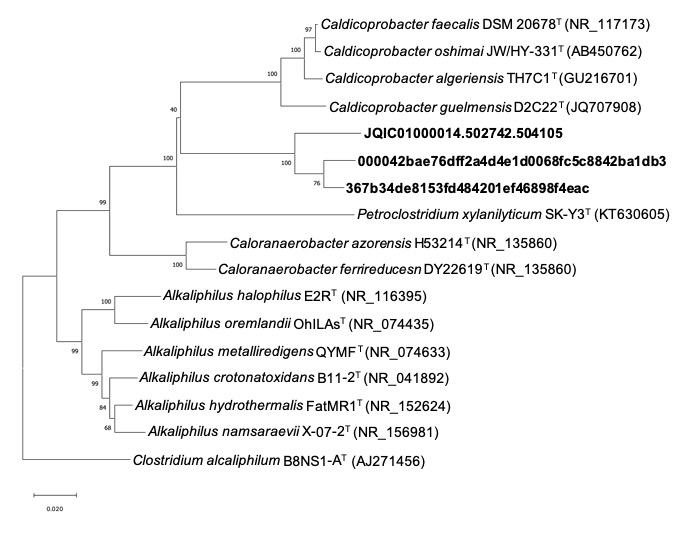
 **Supplementary Figure 4:** Neighbour-joining phylogenetic tree reconstructed from the type strains of genera in the Clostridiaceae family that are closley related to the representative sequences classified as representing the *Caldicoprobacter* genus in the 16S rRNA gene amplicon analysis. Bootstrap values >40 %, based on 1000 replicates, are indicated on branch points. *Clustridium* *alcaliphilum* was used as an outgroup. Bar, 0.02 nucleotide substitutions per site.
